# CAR-NK cells for cancer immunotherapy: recent advances and future directions

**DOI:** 10.3389/fimmu.2024.1361194

**Published:** 2024-02-09

**Authors:** Tianye Li, Mengke Niu, Weijiang Zhang, Shuang Qin, Jianwei Zhou, Ming Yi

**Affiliations:** ^1^ Department of Gynecology, The Second Affiliated Hospital, Zhejiang University School of Medicine, Hangzhou, China; ^2^ Zhejiang Provincial Clinical Research Center for Obstetrics and Gynecology, Hangzhou, China; ^3^ Department of Oncology, Tongji Hospital of Tongji Medical College, Huazhong University of Science and Technology, Wuhan, China; ^4^ Department of Radiation Oncology, Hubei Cancer Hospital, Tongji Medical College, Huazhong University of Science and Technology, Wuhan, China; ^5^ Department of Breast Surgery, The First Affiliated Hospital, College of Medicine, Zhejiang University, Hangzhou, China

**Keywords:** natural killer cell, CAR-NK, cancer immunotherapy, the tumor microenvironment, adoptive cell transfer

## Abstract

Natural Killer (NK) cells, intrinsic to the innate immune system, are pivotal in combating cancer due to their independent cytotoxic capabilities in antitumor immune response. Unlike predominant treatments that target T cell immunity, the limited success of T cell immunotherapy emphasizes the urgency for innovative approaches, with a spotlight on harnessing the potential of NK cells. Despite tumors adapting mechanisms to evade NK cell-induced cytotoxicity, there is optimism surrounding Chimeric Antigen Receptor (CAR) NK cells. This comprehensive review delves into the foundational features and recent breakthroughs in comprehending the dynamics of NK cells within the tumor microenvironment. It critically evaluates the potential applications and challenges associated with emerging CAR-NK cell therapeutic strategies, positioning them as promising tools in the evolving landscape of precision medicine. As research progresses, the unique attributes of CAR-NK cells offer a new avenue for therapeutic interventions, paving the way for a more effective and precise approach to cancer treatment.

## 1 Background

Natural Killer (NK) cells are essential contributors to the immune response, demonstrating formidable cytotoxic capabilities towards infected, stressed, transformed, and foreign cells (1). Belonging to the innate lymphoid cells (ILCs) family, NK/cells actively engage in the initial stages of host defense. They originate from CD34^high^ hematopoietic stem cells (HSCs) in the bone marrow (2). The developmental stages involve differentiation from HSCs toward common lymphoid progenitors, progressing to NK cell progenitors. Although the bone marrow serves as the primary site for NK cell development, extramedullary maturation takes place in secondary lymphoid tissues, including the thymus and lymph nodes. Mature NK cells originating in the bone marrow migrate towards secondary lymphoid tissues and peripheral organs (3). The maturation process is marked by changes in surface markers, with CD56 expression indicating distinct functional properties (4). CD56^bright^ NK cells are primarily cytokine releasers, evolving into cytotoxic CD56^dim^ NK cells with further maturation, acquiring type III Fcγ receptor CD16 and killer-cell immunoglobulin-like receptors (KIRs) (5). Memory-like features in NK cells are observed following exposure to cytokines like IL-12, IL-15, and IL-18. These memory-like NK cells exhibit improved activation upon re-exposure to stimulation, contributing to adaptive immune responses (6–10).

Mature NK cells undergo orchestrated migration from the bone marrow to SLTs and various peripheral non-lymphoid organs. This migration is regulated by a myriad of molecules, such as chemokines, integrins, and selectins. The receptors S1P5 and CX3CR1, along with downregulated CXCR4, facilitate NK cell exit from bone marrow into the bloodstream (11). Specific receptors and ligands dictate NK cell homing to organs. For example, CCR7 engagement with CCL19 and CCL21 is crucial for NK cell homing to lymph nodes (12). NK cells are found in various organs, each exhibiting distinct trafficking patterns. The liver, lungs, intestine, uterus, and even the central nervous system (CNS) are infiltrated by NK cells, contributing to immune surveillance (13, 14). The local environment of liver involves both resident and recruited NK cells, promoting tolerance to avoid chronic inflammation. Chemokine receptors like CXCR6 play an important role in NK cell retention in the liver, and similar mechanisms guide NK cell infiltration in other organs, including the decidua and lungs (15–17).

NK cells contribute significantly to the defense mechanisms of the host by directly targeting and eliminating infected and transformed cells (18, 19). Additionally, they release cytokines that coordinate the actions of various immune subsets. Importantly, the killing mechanism employed by NK cells is distinct from that of adaptive T lymphocytes, as it operates without being constrained by human leukocyte antigen (HLA) restriction (20). The balance between suppressive and activating receptors on NK cells determines their status, with inhibitory receptors recognizing major histocompatibility complex (MHC) class I molecules on target cells (21). In the context of cancer biology, NK cells exhibit anti-tumor activity, targeting cells with low MHC-I expression and recognizing stress-induced ligands. Inhibitory receptors, including killer cell immunoglobulin-like receptors (KIRs) and CD94/NKG2A, contribute to NK cell education, ensuring self-tolerance (22). Activating receptors, such as NK gene 2D (NKG2D) and natural cytotoxicity receptors (NCRs), play crucial roles in NK cell responses against cancer cells (23, 24). NK cells can also contribute to cancer therapy through antibody-dependent cell-mediated cytotoxicity (ADCC) effects by CD16 receptors (25–27). The activity of NK cells is subject to intricate regulation by a balance of inhibitory and activating receptors, which together play a crucial role in the immune response of NK cells against tumors and foreign pathogens. This delicate equilibrium ensures that NK cells can discriminate between healthy cells and those presenting abnormalities, such as infected or malignantly transformed cells, contributing significantly to the body’s natural immune surveillance mechanisms. This comprehensive overview provides a nuanced understanding of NK cell development, particularly in cancer progression and therapeutic interventions. The integrated perspective highlights the multifaceted roles of NK cells in immune surveillance and their potential as therapeutic targets in cancer treatment.

## 2 The role of NK cells in cancer progression

NK cells, play a crucial role in the complex landscape of cancer development (28). Specifically implicated in hematopoietic tumors, these cells engage in direct interactions with tumor cells, marking them as promising candidates for therapeutic interventions in the battle against cancers (29). The significance of NK cells in cancer immunotherapy is underscored by their unique ability to recognize and eliminate abnormal cells without prior sensitization, a feature that sets them apart from other immune cells (29). Despite their potential, the practical application of NK cells in cancer therapy faces a myriad of challenges. One prominent hurdle is the limited infiltration of NK cells into solid tumors, a phenomenon that stands in stark contrast to the robust infiltration of cytotoxic T lymphocytes (CTLs) (30, 31). This discrepancy raises questions about the mechanisms that impede the effective interaction between NK cells and the tumor microenvironments (TME). Understanding these impediments is crucial for optimizing NK cell-based therapeutic strategies.

A multifaceted obstacle to NK cell infiltration lies in the intricate architecture of the TME. The solid-tumoral contact and subsequent infiltration of NK cells are thwarted by various factors, contributing to a significantly lower density of infiltrated NK cells when compared to CTLs (31). The hypoxic conditions within the TME, characterized by increased levels of hypoxia-inducible factor 1 alpha (HIF-1α), emerge as a significant player in blunting NK cell activity against solid tumors (32). HIF-1α not only contributes to the immunosuppressive milieu but also hampers the efficacy of NK cell-mediated anti-tumor responses (32). Adding another layer of complexity to the interaction between NK cells and tumors, recent research has provided insights into the role of cancer-derived exosomes in undermining NK cell function (33). These extracellular vesicles, secreted by cancer cells, carry bioactive molecules that can modulate the immune response. The impact of cancer-derived exosomes on NK cells further emphasizes the need for a comprehensive understanding of the intricate crosstalk within the TME (33).

NK cells face the challenge of overcoming these obstacles and successfully infiltrate solid tumors. First, they must extravasate from the bloodstream, navigating through the stiff extracellular matrix (ECM) and tumor stroma. This process involves the secretion of enzymes such as urokinase plasminogen activator, matrix metalloproteinases, and serine dipeptidyl peptidase IV (34). Additionally, heparinase plays a crucial role in this process, emphasizing the complexity of the microenvironment that NK cells must navigate (35). However, even when NK cells overcome these physical barriers and reach the tumor site, their journey is far from over (36, 37). The TME, molded by various immunosuppressive factors, transforms arriving NK cells into a state of immunosuppression. This transformation involves exposure to a plethora of immunosuppressive cytokines, including transforming growth factor-beta (TGF-β), activin-A, adenosine, IL-10, and prostaglandin E2 (PGE2) (38–43). Additionally, the TME harbors inhibitory immune cells that further hamper the functions of NK cell within solid tumors (44–47).

Despite these challenges, the infiltration of NK cells into the TME is associated with a relatively favorable and promising prognosis in numerous cancers (48–50). This observation highlights the role of NK cells in cancer initiation and progression, while facing formidable barriers, their presence within the TME signifies a potential positive outcome. Mouse models provide further evidence of anti-tumor capabilities, revealing that NK cells actively suppress tumor occurrence through immunosurveillance (51–53). Understanding the mechanism of NK cell activation is fundamental to unraveling their role in tumor occurrence and development. The activation process within the TME involves a complex interplay of activating and inhibiting receptors, along with various cytokines and their corresponding ligands or receptors (54–56). These receptors allow NK cells to specifically recognize biomarkers presented on the cancer cell membrane, setting the stage for either tumor surveillance or the promotion of tumor immune escape.

The activation of NK cells culminates in their ability to eliminate tumor cells through multiple mechanisms. Direct release of perforin and granzymes, as well as the induction of apoptosis through ADCC effects, Fas ligand (FasL), or TNF-related apoptosis-inducing ligand (TRAIL), represents the tumor-killing toolkit of activated NK cells. Furthermore, the secretion of lymphokines, including IFN-γ and TNF-α, contributes to retard tumor growth across various cancer types (28). An intriguing facet of NK cell-mediated anti-tumor responses is the release of neoantigens upon the destruction of tumor cells. These neoantigens act as beacons, prompting an adaptive immune response that extends beyond the immediate actions of NK cells. This communicative bridge between innate and adaptive immunity involves dendritic cells (DCs), which play crucial roles in tumor immune responses. NK cells, in their activated state, promote the recruitment of conventional DCs to the TME, further enhancing the immune elimination of tumor cells (57).

While hurdles exist in their efficient infiltration into solid tumors and the subsequent immunosuppressive shaping by the TME, the potential for NK cells to act as potent tumor suppressors cannot be overlooked (58–60). Advancements in understanding the intricacies of NK cell activation, the complex TME, and innovative approaches like iPSC-derived NK cells collectively pave the way for novel therapeutic strategies (61–65). Harnessing the full potential of NK cells holds the promise of revolutionizing cancer treatment, particularly in the context of “cold” tumors that lack neoantigens, thereby surpassing conventional cancer therapies and ushering in a new era of precision immunotherapy (66).

## 3 Chimeric antigen receptor-engineered natural killer cells in cancer immunotherapy

In the realm of cancer treatment, immunotherapy has emerged as a revolutionary approach, particularly with the advent of Chimeric Antigen Receptor (CAR) technology. The emergence of CAR-NK cell therapy represents a promising shift in the landscape of cancer immunotherapy, offering potential solutions to the challenges faced by CAR-T cell therapy (67). While CAR-T cells have demonstrated clinical benefit in some specific hematological cancers, the field has encountered obstacles, including the time-intensive generation of therapeutic doses and the difficulty in obtaining sufficient autologous T cells from heavily pre-treated cancer patients (68–70). In response to these challenges, CAR-NK cell therapy emerges as an attractive alternative, showcasing distinct advantages (71).

One of the primary strengths of CAR-NK cells lies in their use of allogeneic NK sources, addressing concerns related to GVHD. Unlike CAR-T cells, CAR-NK cells can utilize an unlimited allogeneic NK source without triggering GVHD, providing a considerable safety advantage (72). Furthermore, CAR-NK therapy introduces the potential for “off-the-shelf” products, leveraging NK cell lines or iPSC-NK, resulting in a substantially shortened production time (73, 74). This efficiency is crucial for treating patients with rapidly progressing diseases, a limitation often encountered in CAR-T therapy. The versatility of CAR-NK cells is evident in their ability to target a broad range of tumor antigens in both hematological and solid malignancies (61, 75–82). The antigen recognition domain, typically consisting of a single-chain fragment derived from a monoclonal antibody, facilitates targeted therapy. While CD19/CD20/CD33 remain major targets for hematological cancers, CAR-NK cells have demonstrated efficacy against solid cancer targets, such as Her2, EpCAM, and EGFR, offering a broader spectrum of applicability (82–85).

**Figure 1:**
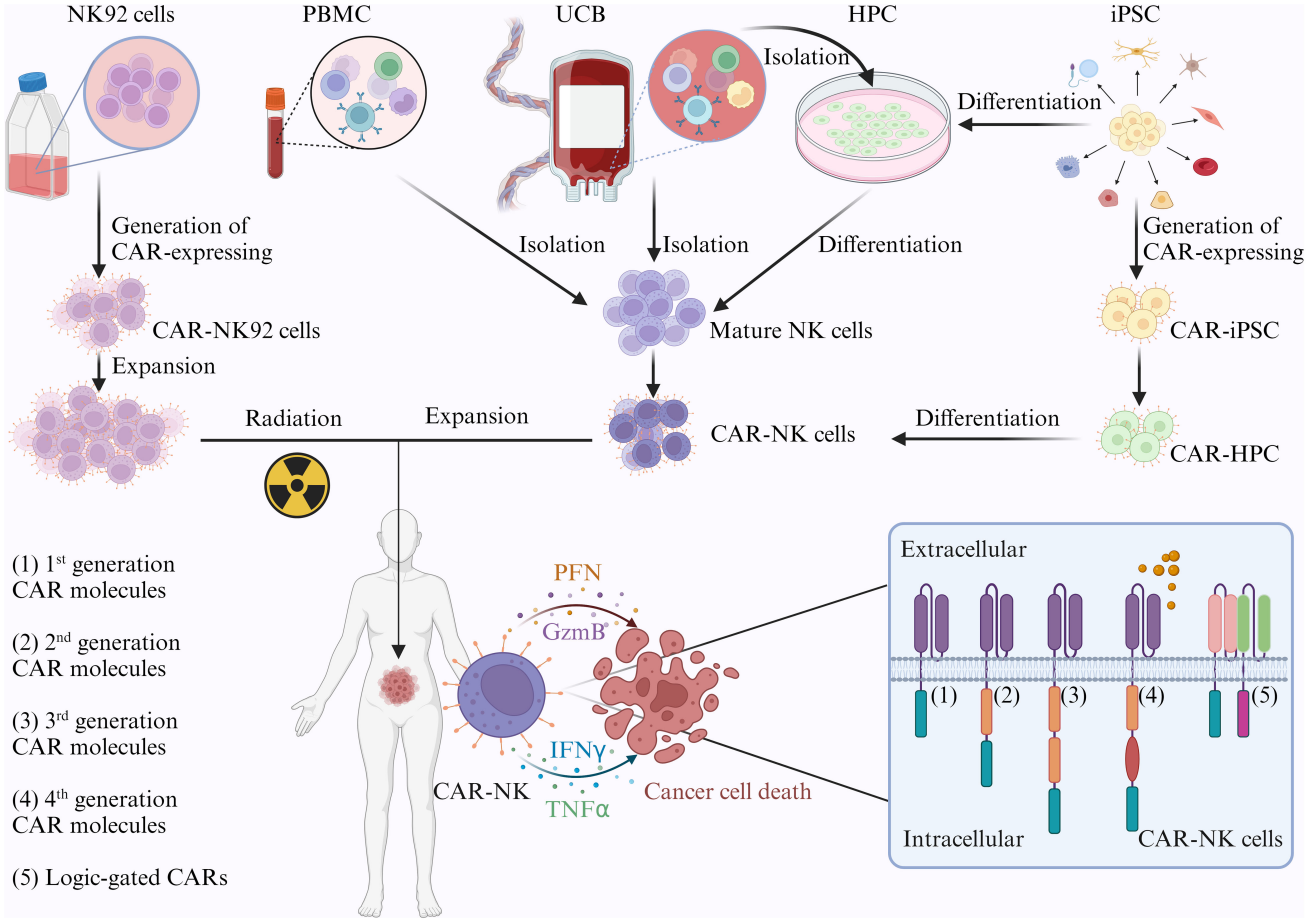
The development of CAR-NK cells. The NK92 cell line is commonly used due to its ability to indefinitely expand *in vitro*. Primary NK cells can be directly isolated from peripheral blood mononuclear cells (PBMCs) or umbilical cord blood (UCB) using a NK cell isolation kit. These cells are then activated, genetically modified with CAR-expressing vectors, and expanded in NK cell-specific media with cytokines for clinical use. CD34+ hematopoietic progenitor cells (HPCs) can be differentiated into NK cells with a cytokine cocktail, and these cells are engineered with CAR before *in vitro* expansion and infusion. Induced pluripotent stem cells (iPSCs) have emerged as a promising “off-the-shelf” source for CAR-NK cells, given their unlimited proliferative capacity. iPSCs can differentiate into CD34+ HPCs, then into NK cells. Importantly, CAR-expressing vectors can be introduced into iPSCs, leading to CAR-iPSCs, which can further differentiate into CAR-HPCs and CAR-NK cells (Created with Biorender).

Like CAR-T cells, CAR-NK cells undergo genetic modification to express CARs designed to recognize specific antigens present on target cells (**Figure 1**). The pre-clinical studies have employed various delivery methods, such as lentiviral or retroviral-based transduction, transposon systems, and electroporation of mRNA, highlighting the adaptability of CAR-NK technology (86). The signaling domains of CAR-NK cells closely resemble those of CAR-T cells, incorporating TCR co-stimulatory molecules, such as CD28, 4-1BB, NKG2D, 2B4, and DNAM1 (86). Notably, CAR-NK cells not only direct cytotoxicity against tumor cells by targeting specific antigens but also exhibit potential in eliminating immunosuppressive cells within the TME. This includes targeting myeloid-derived suppressor cells (MDSCs) and M2-like tumor-associated macrophages (TAMs), indicating a broader therapeutic impact beyond direct tumor cell killing (87, 88). The proposed combination of CAR-NK cells with T cell-based therapies for solid tumors further underscores their potential in reshaping cancer immunotherapy strategies. The safety profile of CAR-NK cells is a significant advantage, with allogeneic haploidentical NK cells demonstrating safety for adoptive cell therapy by reducing the risk of GVHD (89). Unlike CAR-T cells, CAR-NK cells exhibit fewer safety concerns, including on-target/off-tumor effects, cytokine release syndrome (CRS), and tumor lysis syndrome. The unique ability of CAR-NK cells to detect MHC class I-negative tumor cells further extends their applicability (90).

CAR-NK cell-mediated immunotherapy has rapidly emerged as a compelling alternative for patients facing metastatic malignancies, showcasing considerable promise in the realm of cancer immunotherapy (91). Despite significant exploration of CAR-NK cells in preclinical studies, their applications in various tumor models are predominantly at the preclinical stage, showing both potential and the need for further investigation. Notably, clinically approved second-generation CAR-NK cells, incorporating the CD3ζ domain and a 4-1BB or CD28 co-stimulatory domain, have primarily targeted CD19+ lymphoid-derived hematologic malignancies (92).

Initially designed to combat hematological malignancies such as lymphoma, myeloma, and leukemia, CAR-NK cells have demonstrated efficacy in preclinical settings. CD19-CAR-NK cells, in particular, have exhibited superior efficiency in treating lymphoid malignancies compared to CAR-T-based cellular immunotherapy, showcasing their potential advantages (92). For instance, the phase 1/2 study NCT03056339 explored the efficacy of anti-CD19 CAR-NK cells, derived from cord blood, in 11 patients with relapsed or refractory CD19+ hematologic cancers (93). The CAR-NK cells, modified with a safety switch, were administered in varying doses after lymphodepleting chemotherapy (93). Results showed that CAR-NK cell treatment was well-tolerated, with no major toxic effects such as cytokine release syndrome or neurotoxicity (93). Of the 11 patients, 73% responded positively to the treatment, with 7 achieving complete remission (93). Responses were rapid, observed within 30 days, and CAR-NK cells persisted for at least 12 months. The study suggests the potential effectiveness and safety of CAR-NK cell therapy in CD19+ cancers (93).

Beyond hematological malignancies, CAR-NK cells hold promise for addressing metastatic solid tumors, where CAR-T cell therapy faces substantial limitations (94). With intrinsic advantages such as substantial cytolytic ability, non-MHC-restricted recognition, natural tumor tissue infiltration, and minimal untoward effects, CAR-NK cells present a viable therapeutic option for solid tumor management (94). Preclinical studies have demonstrated their efficacy against diverse solid tumors (95, 96). However, clinical data on CAR-NK cells in solid tumor treatment remains limited, with ongoing phase I/II trials showing feasibility and potential efficacy. Continued exploration and clinical trials are essential to unravel the safety and efficacy of CAR-NK cells in the dynamic landscape of cancer therapy. Additionally, Generally, CAR-NK cell therapy stands out as a promising alternative to CAR-T therapy, presenting a myriad of advantages, including safety, versatility, and efficiency. Current clinical trials, examining the safety and effectiveness of CAR-NK cell therapy in both hematological and solid malignances, underscore its potential to transform the landscape of cancer treatment (**Table 1**). Further research and clinical exploration are crucial to fully understand and harness the transformative power of CAR-NK cells in the realm of cancer immunotherapy.

**Table 1:** The ongoing clinical trials of CAR-NK in cancer immunotherapy, which have progressed beyond phase 1 thus far, are documented.

CAR target	NK cell source	Targeting tumor	NCT number
CD19	UCB	Hematological malignancies	NCT03056339
	Non-referred	B cell hematologic malignancies	NCT05570188
	HPCs	B-cell lymphoma	NCT05654038
CD70	UCB	Hematological malignancies	NCT05092451
	UCB	Solid tumors	NCT05703854
CD19/CD70	UCB	B-cell NHL	NCT05842707
CD19/CD28	UCB	B-cell NHL	NCT03579927
CD5	UCB	Hematological malignances	NCT05110742
CD7	PBMCs	Leukemia and lymphoma	NCT02742727
CD123	PBMCs	AML and BPDCN	NCT06006403
PD-L1	NK92	GEJ cancers or HNSCC	NCT04847466
Claudin6	PBMCs	Reproductive system tumors	NCT05410717
BCMA	NK92	MM	NCT03940833
CD33	NK92	AML	NCT02944162
MUC1	PBMCs	Solid tumors	NCT02839954
Robo1	NK92	Pancreatic cancer	NCT03941457
TROP2	UCB	Ovarian cancer, mesonephric-like adenocarcinoma, and pancreatic cancer	NCT05922930

UCB, Umbilical cord blood.

HPCs, Hematopoietic progenitor cells.

NHL, Non-Hodgkin lymphoma.

PBMCs, Peripheral blood mononuclear cells.

AML, Acute myeloid leukemia.

BPDCN, Blastic plasmacytoid dendritic cell neoplasm.

GEJ, Gastroesophageal junction.

HNSCC, Head and neck squamous cell carcinoma.

MM, Multiple myeloma.

There are some other cancer immunotherapy paradigms except CAR strategy, such as oncolytic viruses and immune checkpoint inhibitors (97, 98). CAR-NK cells, oncolytic viruses, and immune checkpoint inhibitors represent cutting-edge cancer therapies with distinct mechanisms and applications. CAR-NK cells, engineered to target specific cancer antigens, are notable for their safety and specificity, particularly in hematologic malignancies. Oncolytic viruses selectively infect and kill cancer cells, showing promise in solid tumors; however, their effectiveness varies (99). Immune checkpoint inhibitors, enhancing the immune system’s ability to fight cancer, have been successful across various cancer types but can cause significant immune-related side effects. While CAR-NK cells are in early research stages and involve complex manufacturing, oncolytic viruses require careful genetic engineering for effectiveness. Immune checkpoint inhibitors, widely applicable, face challenges in cost and accessibility. Each therapy offers unique benefits and limitations, shaping individual treatment plans based on cancer type, stage, and patient health. The evolving landscape of these therapies continues to advance cancer treatment options.

## 4 Perspective and conclusion

In conclusion, the intricate interplay between NK cells and the formidable challenges posed by the TME highlights the dualistic nature of these immune actors in the context of cancer. While obstacles such as limited infiltration into solid tumors and the immunosuppressive milieu within the TME impede the full realization of NK cell potential, their presence within tumors is associated with a relatively favorable prognosis. Understanding the complex activation processes involving key receptors and cytokines is pivotal for unraveling their role in tumor occurrence and development. Advancements, particularly in iPSC-derived NK cells, offer new dimensions to NK cell-based immunotherapy, potentially revolutionizing cancer treatment by enhancing tumor surveillance, elimination functions, and the recruitment of T cells to the TME.

Simultaneously, the emergence of CAR-NK cell therapy presents a dynamic and promising alternative in the realm of cancer immunotherapy. The unique advantages of CAR-NK cells, including allogeneic sourcing, reduced risk of GVHD, and innate anti-tumor capabilities, position them as a versatile tool in the fight against cancer. While preclinical successes demonstrate their efficacy against hematological and solid malignancies, addressing challenges such as low cell persistence and efficient trafficking to tumor sites is imperative for successful clinical integration. CAR-NK cell therapy, additionally, faces challenges including limited NK cell persistence and difficulties in targeting and infiltrating solid tumors due to the complex tumor microenvironment. These factors impact CAR-NK cells’ effectiveness, particularly in solid tumor treatments​. The ongoing clinical trials targeting various antigens underscore the commitment to realizing the therapeutic potential of CAR-NK cells, marking a critical phase in reshaping the landscape of cancer treatment.

In navigating the future of cancer immunotherapy, the convergence of NK cells and CAR-NK cells offers a transformative outlook. The intricate mechanisms underlying NK cell activation and the adaptability of CAR-NK cells in recognizing diverse tumor antigens illuminate the depth of research required for their successful integration into mainstream oncology. As these cellular therapies progress from scientific promise to clinical reality, they hold the potential to redefine precision immunotherapy, providing renewed hope and improved outcomes for patients facing challenging malignancies. In this evolving landscape, NK cells and CAR-NK cells stand as promising agents, guiding us toward a future where cancer treatment is not just a battle but a personalized, targeted, and effective therapeutic strategy.

## Author contributions

MY: Conceptualization, Funding acquisition, Supervision, Writing – review & editing. TL: Writing – original draft. MN: Writing – review & editing. WZ: Writing – review & editing. SQ: Writing – review & editing. JZ: Supervision, Writing – review & editing.
